# Whether weekend warriors (WWs) achieve equivalent benefits in lipid accumulation products (LAP) reduction as other leisure-time physical activity patterns? -Results from a population-based analysis of NHANES 2007–2018

**DOI:** 10.1186/s12889-024-19070-z

**Published:** 2024-06-09

**Authors:** Wei Dai, DongYang Zhang, ZhiYuan Wei, Pan Liu, QianKun Yang, Li Zhang, Jie Zhang, Chao Zhang, Hao Xue, Zhao Xie, Fei Luo

**Affiliations:** 1 National & Regional United Engineering Lab of Tissue Engineering, Department of Orthopedics, Southwest Hospital, Third Military Medical University (Army Medical University), Chongqing, 400038 China; 2 Department of Neurosurgery, National Clinical Research Center for Child Health and Disorders, Ministry of Education Key Laboratory of Child Development and Disorders, Chongqing Key Laboratory of Pediatrics, Children’s Hospital of Chongqing Medical University, Chongqing, 400014 China; 3 Department of Orthopedics, The 75th Group Army Hospital of the PLA, Dali, 671003 China; 4 Department of Orthopaedics, Southwest Hospital, Third Military Medical University (Army Medical University), No.29 Gaotanyan St., Shapingba District, Chongqing, 400038 China

**Keywords:** Obesity, Weekend warriors, Regularly active, Lipid accumulation products, Physical activity

## Abstract

**Background:**

Obesity is characterized by excessive fat accumulation in the body. Physical activity (PA) is an effective intervention to combat obesity, but the effectiveness of different PA patterns on controlling obesity is unclear. Lipid accumulation product (LAP), derived from waist circumference and triglycerides, is a novel indicator for obesity evaluation. However, the association between PA patterns (i.e., weekend warriors and regularly active) and LAP remains unexplored. This study aims to elucidate the relationship between PA patterns and LAP in US adult population.

**Methods:**

Adult individuals with complete data on LAP, PA patterns, and other covariates from the National Health and Nutrition Examination Survey (NHANES) database (2007–2018) were included in this study. Multivariate linear regression models were utilized to explore the association between PA patterns and LAP. Subgroup analyses, interaction tests, restricted cubic spline (RCS) regression analyses, and threshold and saturation effect analyses were also performed to investigate the stability and nonlinearity of PA-LAP association, respectively.

**Results:**

A total of 11,212 participants were included in this study. After adjusting for all potential covariates, being regularly active (RA) (β=-8.85, *P* < 0.05) obtained significantly higher LAP reduction as opposed to being weekend warriors (WWs) (β=-4.70, *P* = 0.3841). Furthermore, subgroup analyses and interaction tests indicated that the PA-LAP association was more pronounced in individuals with higher education levels (P interaction = 0.0084) and diabetes (P interaction = 0.0062). Additionally, a significant, non-linear, and negative correlation between weekly total PA and LAP in non-inactive individuals was identified by RCS analysis (P for overall < 0.001, P for nonlinearity = 0.009). A threshold of 440 min in weekly total PA was found to arouse favorable LAP reduction.

**Conclusions:**

Being regularly active obtained better LAP reduction as opposed to being WWs. For non-inactive adults, engaging in more than 440 min of PA per week helps to reduce LAP effectively.

**Supplementary Information:**

The online version contains supplementary material available at 10.1186/s12889-024-19070-z.

## Introduction

Obesity is a multifactorial disease characterized by excess body fat accumulation, which is tightly linked to various unfavorable health outcomes, including hypertension [1], diabetes [2], cardiovascular diseases (CVD) [2], cancers [3], etc. Since 1980, the global obesity prevalence has surged significantly and continues to escalate rapidly [4], making it a serious public health problem worldwide. Therefore, studies concentrating on unraveling obesity pathogenesis, identifying evaluation biomarkers, or developing effective obesity management strategies are urgently needed now.

Since the harm of obesity is primarily derived from metabolic disturbance, including insulin resistance, dyslipidemia, glucose intolerance, etc [5]., a stable and reliable obesity reflective indicator is the pivotal prerequisite for developing obesity interventional strategies. Previous studies have indicated that obese individuals without metabolic abnormalities (namely metabolically healthy/normal obesity) do not get additional risks for suffering cardiometabolic diseases [6]. Therefore, metabolic indicators should be included in establishing obesity reflective indicators. Besides, significant changes in body anthropometrics usually occurred among obese individuals, such as waist circumference, sagittal abdominal diameter, BMI, and so on [7]. Given this, obesity indicators composed of metabolic and anthropometric indexes may be more reflective for the onset and progression of obesity.

Lipid accumulation products (LAP) is a sex-specific indicator calculated from waist circumference (WC) and triglycerides (TG), which is a pivotal index for various metabolic disorders, especially obesity [8]. Although BMI is an easily-accessible indicator for defining obesity, it fails to evaluate the underlying fat distribution and metabolic disorders of obesity. As opposed to BMI, LAP can provide a more precise evaluation of the extent of fat accumulation, and a higher LAP level usually indicates more severe fat accumulation [9–11]. Additionally, substantial evidences have proved that LAP plays critical roles in various aspects, including diagnosing metabolic syndrome, evaluating risks for diabetes and CVD, serving as key parameter to measure the efficacy of interventions targeted at improving metabolic syndrome [12]. Therefore, LAP can be utilized as a reliable and accurate indicator to detect dynamic alterations of fat accumulation and metabolic disorder in obese individuals.

In parallel with dietary, behavioral, and medication interventions, physical activity (PA) is acknowledged as a“pillar” in combating overweight and obesity [13]. Although a minimum of 150 min of moderate physical activity (MPA), or 75 min of vigorous physical activity (VPA), or equivalent combination of MPA and VPA per week was recommended by World Health Organization to improve cardiovascular health, such levels of weekly total PA are reported to be inadequate to arouse clinically significant weight loss or maintain weight without caloric restriction [14, 15]. Even worse, existing evidences have revealed that approximately 80% of adults and adolescents in the United States failed to meet the recommended minimal level of PA per week (i.e., being insufficiently active) [16]. With the increasing pace of global society, adhering to being regular active (RA) may become challenging for individuals with busy schedules. So, the recent years have witnessed a gradual increase of a new PA pattern, namely weekend warriors (WWs), which means individuals choose to complete at least 150 min of moderate-to-vigorous physical activity (MVPA) within 1 ~ 2 sessions per week [17]. Numerous studies have suggested that being WWs reaps similar health benefits in various aspects as being RA, such as reducing all-cause/ CVD/cancer mortality [18], lowering risks for diabetes [19], mitigating frailty levels [20], alleviating symptoms from depression or psychological distress [21, 22], and so on. However, there is study reporting conflicting results that being WWs (OR, 1.28; 95%CI, 1.02–1.65) obtained significantly higher risks for developing metabolic syndrome compared to being RA [23]. Therefore, even though the beneficial effects of PA has been acknowledged, whether WWs reap equivalent benefits in combating obesity and improving obesity-related metabolic disorders still remains unknown.

Given this, in this study, we mainly focused on exploring the association between different PA patterns and LAP (an obesity indicator) with the dataset from NHANES. Our results may provide PA reference for those obese individuals who are searching their suitable PA patterns.

## Materials and methods

###  Study design and population

**Fig. 1:**
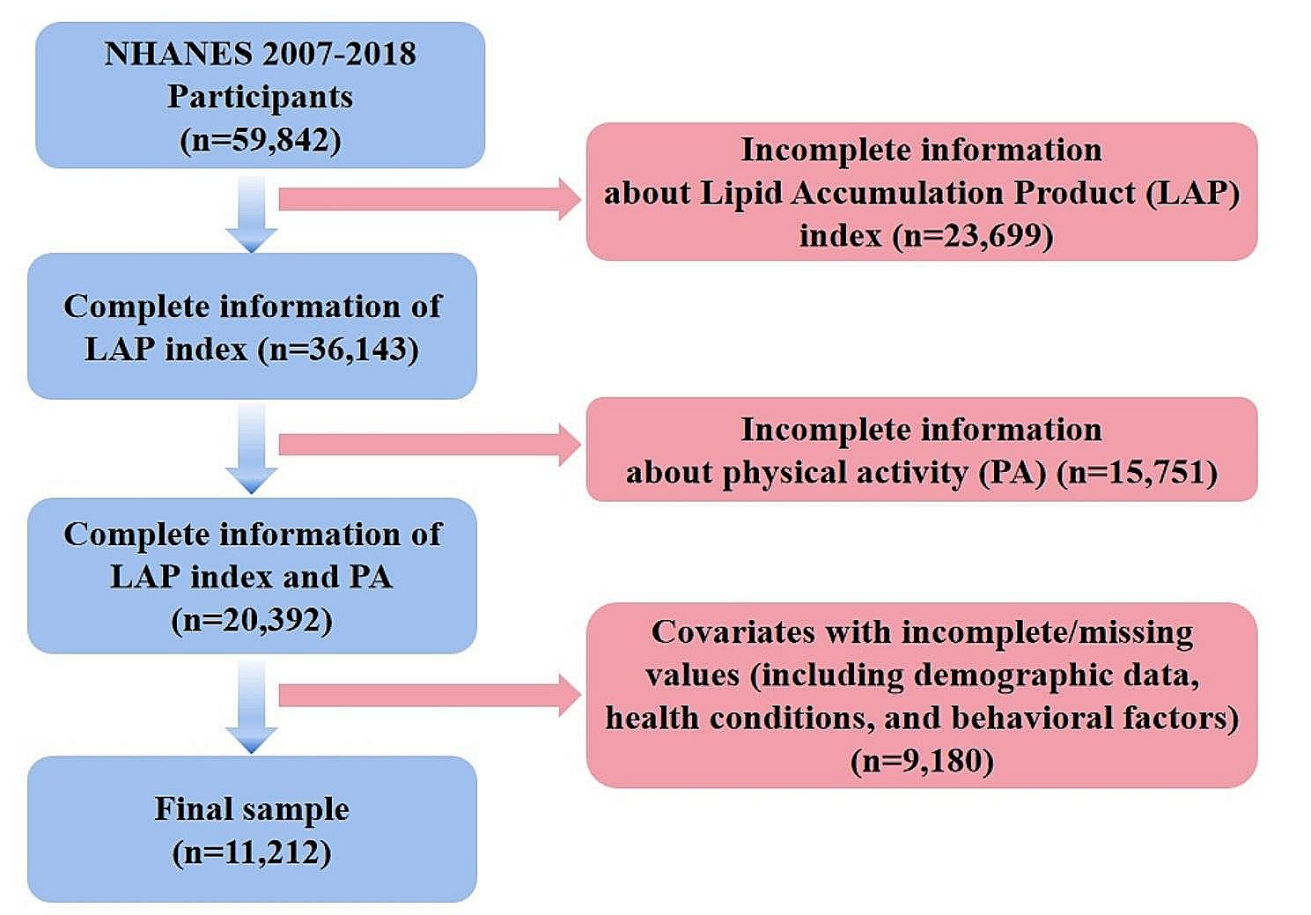
The flowchart of participant selection

The NHANES survey, conducted by the CDC, has been an ongoing investigation of the population since 1999. This study utilizes a complex and stratified probability sampling method, releasing data every two years [24]. Prior to the interviews and examinations, informed consent was obtained from all participants [25]. For this research, we analyzed data from six consecutive NHANES cycles spanning from 2007 to 2008 to 2017–2018, including a total of 59,842 individuals. Adult participants (≥ 20 years old) with incomplete information on body measurements (height, weight, waist circumference, and hip circumference), serum total cholesterol and triglycerides, physical activity, demographic variables (age, gender, race, education, PIR, and marital status), as well as behavioral and health factors (smoking, drinking, CVD, and diabetes) were excluded from the analysis. Ultimately, a total of 11,212 participants was included in the study. The criteria for participants selection in the current study was presented in Fig. 1.

###  Lipid accumulation products (LAPs) assessment

Each participant underwent a household interview and completed a physical examination at the Mobile Examination Center [26]. The measurements of standing height (cm), weight (kg), waist circumference (cm), and hip circumference (cm) were measured using standardized protocols [27]. Blood samples were collected after a fasting period of at least 8 h overnight. Venous blood samples from the participants used for serum total cholesterol and triglycerides (TG) examinations were collected and processed according to the NHANES protocols [28]. The LAP was calculated by using sex-specific formula: LAP=[waist circumference (cm)-65] * TG (mmol/L) for male and [waist circumference (cm)-58] * TG (mmol/L) for female [29].

###  PA and other covariates

**Fig. 2:**
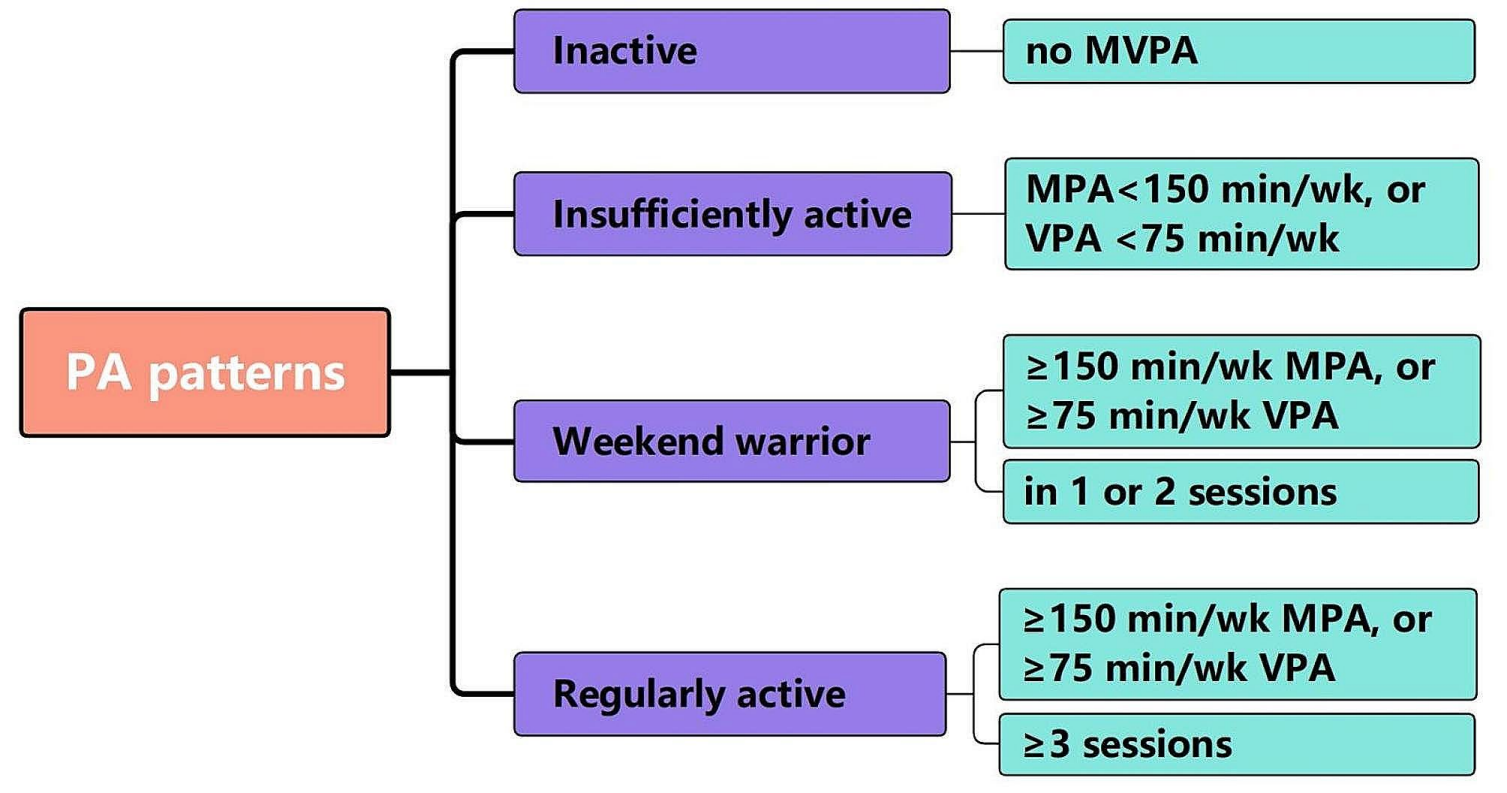
The definition of different physical activity patterns. Abbreviations: MVPA, moderate-to-vigorous physical activity; MPA, moderate physical activity; VPA, vigorous physical activity

PA was assessed by using a PA questionnaire. Participants were asked about the frequency and duration of vigorous and moderate sports, fitness, and recreational activities that lasted for at least 10 continuous minutes in a typical week. PA was calculated by combining the frequency (sessions per week) and duration (duration per time) of these activities. Previous studies have revealed that 1 min of vigorous-intensity activity (VPA) was equivalent to 2 min of moderate physical activity (MPA) [16]. Therefore, total PA was calculated by using the following formula: 2 * vigorous PA + moderate PA. As previously described [18], the activity patterns can be divided into four groups according to self-reported leisure time PA: (1) inactive group (reporting no vigorous or moderate PA), (2) insufficiently active group (reporting less than 150 min per week of total PA), (3) weekend warriors group (reporting at least 150 min of total PA weekly in 1 or 2 sessions), and (4) regularly active group (reporting at least 150 min of total PA weekly in more than 2 sessions). The definition for different PA patterns was presented in Fig. 2.

Based on previous studies, some essential covariates were included in this study, including demographics data, behavioral risk factors, and chronic non-communicable diseases [21, 30, 31]. The demographic covariates included gender, age (≤ 45, 45–65, > 65 years), race/ethnicity (non-Hispanic White, non-Hispanic Black, Hispanic, other), marital status (married/live with partner, divorced/separated/widowed, never married), educational level (less than 9th grade, high school or equivalent, some college or above), and poverty income ratio (PIR) (≤ 1.3, 1.3–3.5, > 3.5). Additional covariates included participants’ behavioral risk factors such as smoking and drinking [32, 33], as well as disease status including diabetes and cardiovascular disease (CVD) [34, 35]. Smoking status was evaluated by the answer to the question of “Have you smoked at least 100 cigarettes in your entire life?”, and those who answered “yes” were defined as smokers, otherwise defined as nonsmokers. Drinking was evaluated by the answer to the question of “Had at least 12 alcohol drinks in any one year?”, and those who answered “yes” were defined as drinkers, otherwise defined as nondrinkers. Body mass index (BMI) was determined using the standard formula (weight/height^2^) based on measured weight (kg) and height (m). Participants were further divided into three groups by BMI based on the criterion from World Health Organization (WHO) [13], including normal or low body weight (< 25 kg/m^2^), overweight (25–30 kg/m^2^), and obese (> 30 kg/m^2^).

###  Statistical analysis

The baseline characteristics of all participants were presented based on the grouping of different PA pattern groups. Variables which are not normally distributed were presented as median (Q1-Q3, interquartile), and categorical variables were presented as numbers and percentages. The differences in baseline characteristics were compared by using Rao-Scott chi-squared test or Kruskal-Wallis test. Multivariate linear regression models were utilized to explore the association between PA patterns and LAP. Additionally, subgroup analyses, interaction tests and restricted cubic spline regression analyses were employed to investigate the robustness and nonlinearity of PA-LAP association, respectively. Furthermore, when a non-linear association between total PA and LAP was determined, a recursive algorithm was employed to compute the inflection point for total PA, and a bi-segmented linear regression model was also established to evaluate the differences before and after the inflection point [36]. Statistical analyses were performed by using R software (version 4.3.1, https://www.r-project.org/). A two-sided *P*-values < 0.05 were considered statistically significant.

## Results

###  Baseline characteristics of the study population

As is shown in Fig. 1, a total of 59,842 participants from 2007 to 2018 were preliminarily included in this study. Participants who had missing data on body measurements (height, weight, waist circumference, and hip circumference), serum total cholesterol and triglycerides, physical activity, demographic variables (age, gender, race, education, PIR, and marital status), as well as behavioral and health factors (smoking, drinking, CVD, and diabetes) were excluded from the analysis. Ultimately, a total of 11,212 participants was included in the study.

The baseline characteristics of participants in different PA pattern groups were presented in Table 1. The sample analyzed consisted of 11,212 participants, including 7,267 inactive individuals, 192 insufficiently active individuals, 122 weekend warriors, and 3,631 regularly active individuals. Participants in different PA pattern groups exhibited significant differences in variables of gender, age, race, educational level, marital status, PIR, diabetes, smoking, drinking, BMI and CVD. Specifically, compared those who in inactive group, participants belonged to WWs group were more likely to be male, aged under 45 years, non-Hispanic White, have higher level of education and PIR, be non-smokers and nondrinkers, and with no CVD or diabetes.

**Table 1:** Participants characteristics according to physical activity patterns

Characteristics	Overall (*n* = 11,212)	Inactive (*n* = 7267)	Insufficiently active (*n* = 192)	Weekend warrior (*n* = 122)	Regularly active (*n* = 3631)	*P*-value
**Gender**						< 0.001
Male	5964 (53.19%)	3743 (51.51%)	96 (50.00%)	98 (80.33%)	2027 (55.82%)	
Female	5248 (46.81%)	3524 (48.49%)	96 (50.00%)	24 (19.67%)	1604 (44.18%)	
**Age**						< 0.001
<=45	5411 (48.26%)	2856 (39.30%)	111 (57.81%)	85 (69.67%)	2359 (64.97%)	
45–65	3963 (35.35%)	2901 (39.92%)	69 (35.94%)	27 (22.13%)	966 (26.60%)	
> 65	1838 (16.39%)	1510 (20.78%)	12 (6.25%)	10 (8.20%)	306 (8.43%)	
**Race**						< 0.001
Mexican American	1661 (14.81%)	1178 (16.21%)	35 (18.23%)	17 (13.93%)	431 (11.87%)	
Other Hispanic	1125 (10.03%)	769 (10.58%)	16 (8.33%)	22 (18.03%)	318 (8.76%)	
Non-Hispanic White	4849 (43.25%)	3162 (43.51%)	64 (33.33%)	54 (44.26%)	1569 (43.21%)	
Non-Hispanic Black	2321 (20.70%)	1519 (20.90%)	33 (17.19%)	16 (13.11%)	753 (20.74%)	
Other Races	1256 (11.20%)	639 (8.79%)	44 (22.92%)	13 (10.66%)	560 (15.42%)	
**Education**						< 0.001
Less than high school	2191 (19.54%)	1859 (25.58%)	32 (16.67%)	19 (15.57%)	281 (7.74%)	
High school or equivalent	2449 (21.84%)	1858 (25.57%)	25 (13.02%)	25 (20.49%)	541 (14.90%)	
Some college or above	6572 (58.62%)	3550 (48.85%)	135 (70.31%)	78 (63.93%)	2809 (77.36%)	
**Marital status**						< 0.001
Married	6222 (55.49%)	4091 (56.30%)	114 (59.38%)	61 (50.00%)	1956 (53.87%)	
Divorced/separated/widowed	2420 (21.58%)	1841 (25.33%)	31 (16.15%)	21 (17.21%)	527 (14.51%)	
Never married	2570 (22.92%)	1335 (18.37%)	47 (24.48%)	40 (32.79%)	1148 (31.62%)	
**PIR**						< 0.001
<=1.3	3128 (27.90%)	2435 (33.51%)	33 (17.19%)	28 (22.95%)	632 (17.41%)	
1.3–3.5	4382 (39.08%)	2870 (39.49%)	82 (42.71%)	52 (42.62%)	1378 (37.95%)	
> 3.5	3702 (33.02%)	1962 (27.00%)	77 (40.10%)	42 (34.43%)	1621 (44.64%)	
**Diabetes**						< 0.001
Yes	1143 (10.19%)	956 (13.16%)	14 (7.29%)	6 (4.92%)	167 (4.60%)	
No	10,069 (89.81%)	6311 (86.84%)	178 (92.71%)	116 (95.08%)	3464 (95.40%)	
**Smoking**						< 0.001
Yes	5239 (46.73%)	3920 (53.94%)	62 (32.29%)	48 (39.34%)	1209 (33.30%)	
No	5973 (53.27%)	3347 (46.06%)	130 (67.71%)	74 (60.66%)	2422 (66.70%)	
**Drinking**						< 0.001
No	8930 (79.65%)	6039 (83.10%)	135 (70.31%)	94 (77.05%)	2662 (73.31%)	
Yes	2282 (20.35%)	1228 (16.90%)	57 (29.69%)	28 (22.95%)	969 (26.69%)	
**BMI**						< 0.001
< 25	3360 (29.97%)	1866 (25.68%)	81 (42.19%)	41 (33.61%)	1372 (37.79%)	
25–30	3635 (32.42%)	2281 (31.39%)	57 (29.69%)	44 (36.07%)	1253 (34.51%)	
>=30	4217 (37.61%)	3120 (42.93%)	54 (28.12%)	37 (30.33%)	1006 (27.71%)	
**CVD**						< 0.001
No	10,452 (93.22%)	6638 (91.34%)	184 (95.83%)	117 (95.90%)	3513 (96.75%)	
Yes	760 (6.78%)	629 (8.66%)	8 (4.17%)	5 (4.10%)	118 (3.25%)	

***Abbreviations:*** PA, physical pattern; LAP, Lipid Accumulation Product; CVD, cardiovascular disease; BMI, body mass index; PIR, ratio of family income to poverty; NHANES, National Health and Nutrition Examination Survey

###  Differences in PA parameters among different PA pattern groups

The characteristics of PA among different PA pattern groups were presented in **Supplementary Table **1. Except for sedentary time, regularly active group exhibited higher levels of PA parameters as opposed to WWs group, insufficiently active group and inactive group, including VPA/MPA sessions per week, VPA/MPA time per session, total VPA/MPA per week, total MVPA and total PA per week (all *P* values < 0.001).

###  Association between PA patterns and LAP

**Fig. 3:**
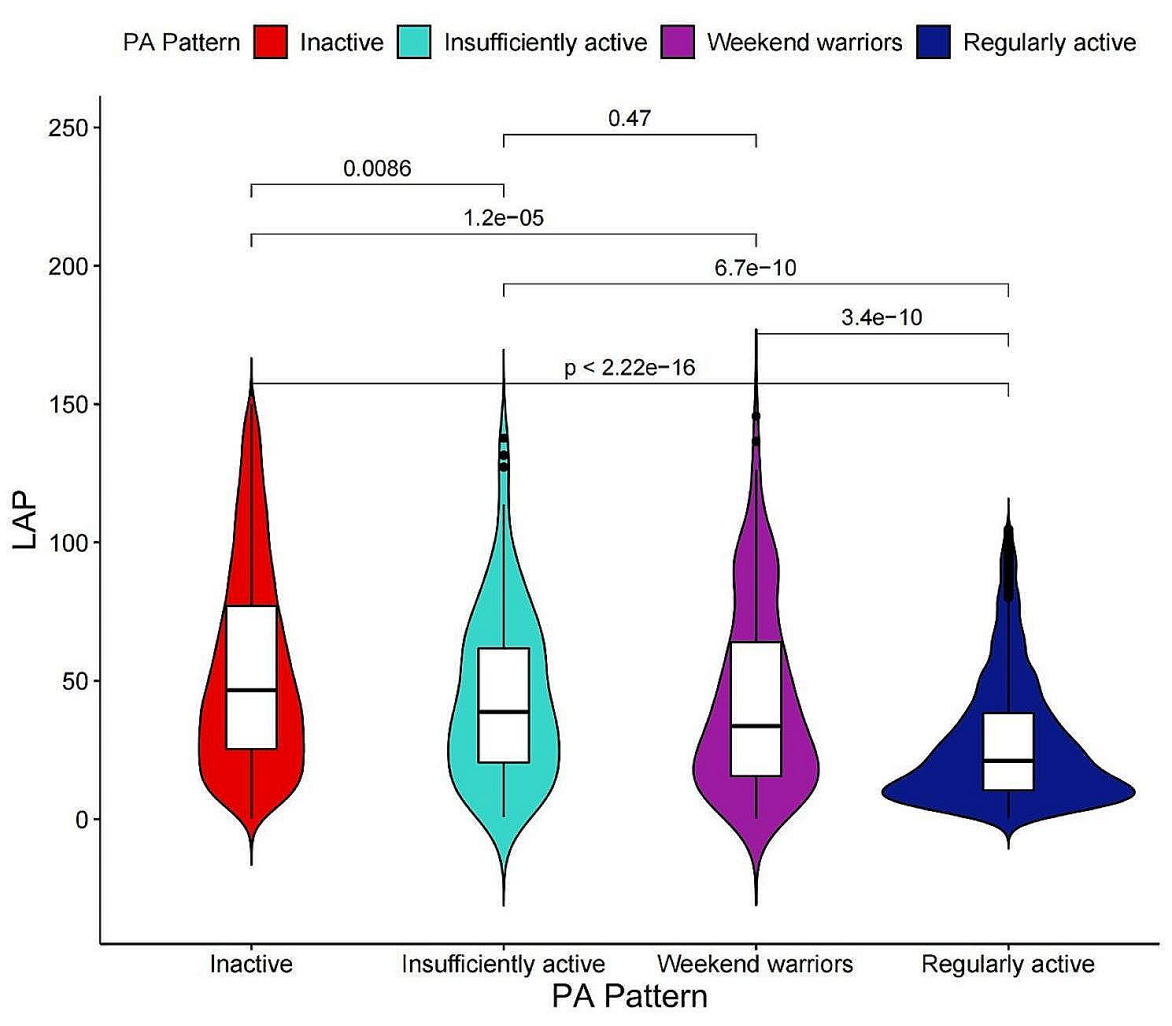
Violin plot of LAP in different PA pattern groups. Abbreviations: PA, physical activity; LAP, lipid accumulation product

In order to clarify the relationship between PA patterns and LAP, the LAP levels between different PA groups were firstly compared. As is depicted in Fig. 3, regularly active individuals obtained significant reduced LAP level as opposed to other three PA pattern groups (*P* < 0.05). Although being insufficiently active or WWs got notable LAP reduction compared with being inactive (*P* < 0.05), no significant differences in LAP reduction were found between being insufficiently active and being WWs (*P* = 0.47).

**Fig. 4:**
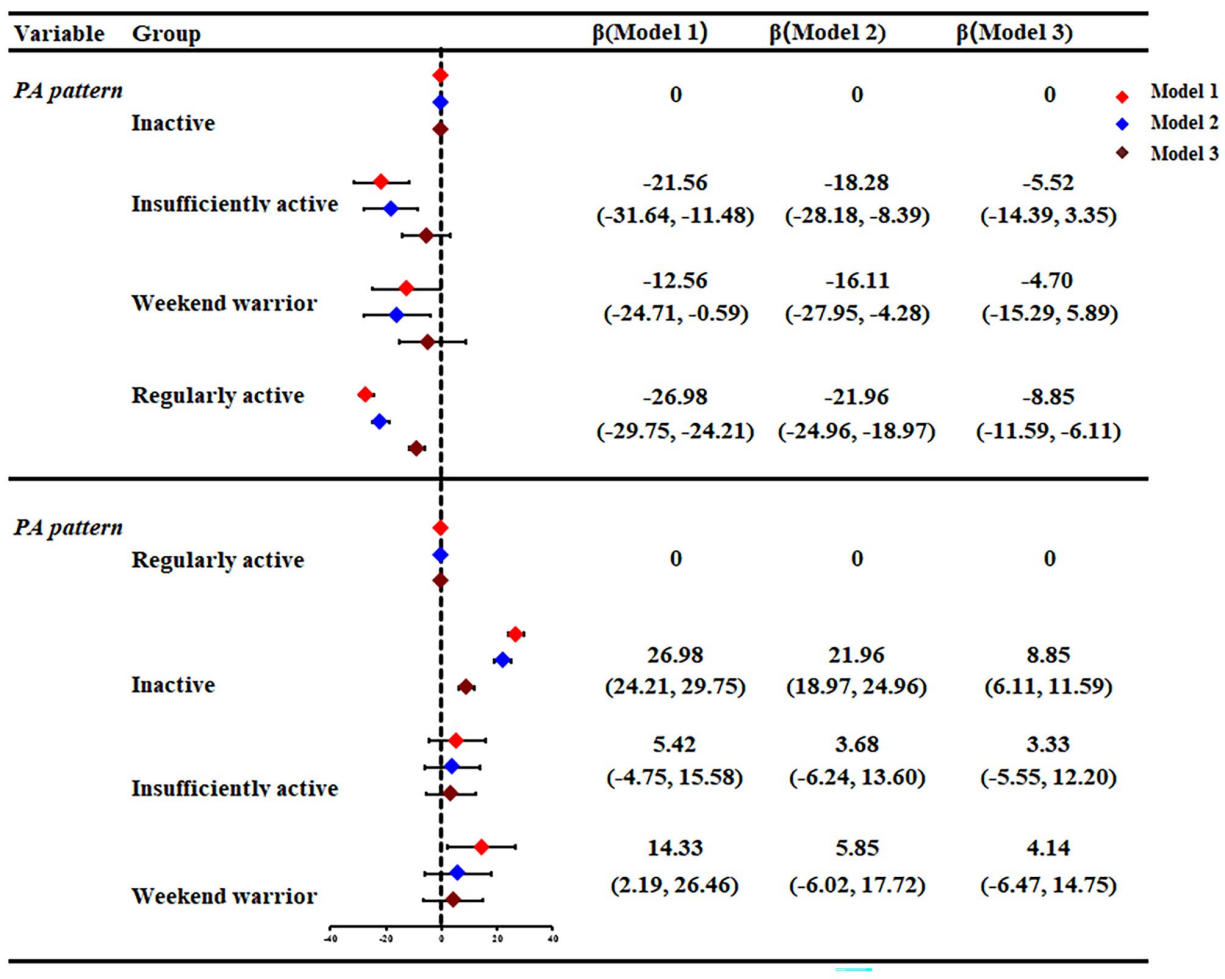
The forest plot of PA patterns and LAP in adults. Abbreviations: PA, physical activity; LAP, lipid accumulation product

Furthermore, multivariate linear regression models were subsequently performed to explore the PA-LAP association, and the results were presented in Tables 2 and Fig. 4. Compared with inactive group, only the regularly active group got significant LAP reduction in non-adjusted model (Model 1, β=-26.98, *P* < 0.0001), partially-adjusted model (Model 2, β=-21.96, *P* < 0.0001) and fully-adjusted model (Model 3, β=-8.85, *P* < 0.0001). Conversely, when the regularly active group was defined as the reference group, being inactive significantly associated with notable LAP increase in all models (Model 1, β = 26.98, *P* < 0.0001; Model 2, β = 21.96, *P* < 0.0001; Model 3, β = 8.85, *P* < 0.0001). Noteworthily, although WWs group didn’t obtain significant LAP reduction as regularly active group, WWs indeed achieve certain LAP reduction, which was reflected by the value of β in all models (β=-12.65, -16.11, -4.70 for model 1, 2 and 3, respectively).

**Table 2:** The relationship between PA patterns and LAP in all participants

Characteristics	Model 1 β (95% CI)	Model 2 β (95% CI)	Model 3 β (95% CI)
**PA pattern**			
Inactive	Reference	Reference	Reference
Insufficiently active	-21.56 (-31.64, -11.48) < 0.0001	-18.28 (-28.18, -8.39) 0.0003	-5.52 (-14.39, 3.35) 0.2227
Weekend warrior	-12.65 (-24.71, -0.59) 0.0398	-16.11 (-27.95, -4.28) 0.0076	-4.70 (-15.29, 5.89) 0.3841
Regularly active	-26.98 (-29.75, -24.21) < 0.0001	-21.96 (-24.96, -18.97) < 0.0001	-8.85 (-11.59, -6.11) < 0.0001
P for trend	< 0.001	< 0.001	< 0.001
**PA pattern**			
Regularly active	Reference	Reference	Reference
Inactive	26.98 (24.21, 29.75) < 0.0001	21.96 (18.97, 24.96) < 0.0001	8.85 (6.11, 11.59) < 0.0001
Insufficiently active	5.42 (-4.75, 15.58) 0.2962	3.68 (-6.24, 13.60) 0.4671	3.33 (-5.55, 12.20) 0.4626
Weekend warrior	14.33 (2.19, 26.46) 0.0207	5.85 (-6.02, 17.72) 0.3339	4.14 (-6.47, 14.75) 0.4442
P for trend	< 0.001	< 0.001	< 0.001

***Abbreviations***: PA, physical pattern; LAP, lipid accumulation product; CI, confidence interval.

Model 1 was the univariate model in which no covariates were adjusted.

Model 2 was adjusted for demographic covariates, including age, gender, race, education, PIR, and marital status.

Model 3, based on Model 2, was additionally adjusted for smoking, CVD, diabetes, alcoholism, and BMI.

Given that the reported mutual associations between PA and diabetes, BMI and CVD, and the correlations between LAP and diabetes, BMI and CVD in previous studies [37–39], potential multicollinearity or collider bias may existed among these variables. Therefore, multivariate linear regression models were further performed without adjusting for the variables of diabetes, BMI and CVD. Our results revealed a similar, consistent and significant trend in LAP reduction between the two regression models with or without adjusting for diabetes, BMI and CVD (Supplementary Table 2).

###  Subgroup analyses

To further confirm the robustness and stability of the association between PA pattern and LAP, subgroup analyses based on grouping of vital covariates were performed. As presented in Table 3, the correlations between PA patterns and LAP remained consistent across different subgroups stratified by the variables of gender, age, race, marital status, PIR, smoking, drinking, CVD and BMI. Additionally, interaction tests revealed that the associations between PA patterns and LAP were modified by the variables of education level and diabetes, individuals being regularly active obtained more pronounced LAP reduction in two subgroups, including individuals with higher education level (P interaction = 0.0084), and with diabetes (P interaction = 0.0062).

**Table 3:** The relationship between PA patterns and LAP in different subgroups

Characteristics	Inactive β (95% CI)	Insufficiently active β (95% CI)	Weekend warrior β (95% CI)	Regularly active β (95% CI)	*P* interaction
**Gender**					0.2840
Male	Reference	-6.84 (-22.48, 8.79) 0.3910	-0.96 (-16.42, 14.50) 0.9028	-10.40 (-15.08, -5.72) < 0.0001	
Female	Reference	5.15 (-3.89, 14.18) 0.2640	-11.41 (-29.17, 6.34) 0.2078	-6.45 (-9.36, -3.54) < 0.0001	
**Age**					0.3160
<=45	Reference	-10.16 (-23.00, 2.67) 0.1206	-4.44 (-19.01, 10.12) 0.5500	-8.54 (-12.56, -4.52) < 0.0001	
45–65	Reference	11.27 (-4.43, 26.97) 0.1596	-1.85 (-26.71, 23.00) 0.8838	-9.77 (-14.95, -4.59) 0.0002	
> 65	Reference	11.21 (-15.13, 37.56) 0.4043	16.16 (-12.66, 44.99) 0.2719	-5.04 (-11.20, 1.12) 0.1088	
**Race**					0.3550
Mexican American	Reference	-26.38 (-48.85, -3.92) 0.0215	28.20 (-3.74, 60.15) 0.0837	-5.66 (-13.46, 2.14) 0.1548	
Other Hispanic	Reference	6.12 (-19.22, 31.46) 0.6361	4.03 (-17.74, 25.81) 0.7167	-9.37 (-16.59, -2.14) 0.0112	
Non-Hispanic White	Reference	5.43 (-13.42, 24.28) 0.5723	-9.85 (-30.36, 10.66) 0.3467	-7.67 (-13.01, -2.33) 0.0049	
Non-Hispanic Black	Reference	9.35 (-5.95, 24.64) 0.2313	-9.26 (-31.08, 12.56) 0.4054	-7.25 (-11.55, -2.95) 0.0010	
Other Races	Reference	1.75 (-12.61, 16.12) 0.8111	5.25 (-20.24, 30.74) 0.6866	-7.92 (-13.76, -2.08) 0.0079	
**Education level**					0.0084
Less than high school	Reference	-0.94 (-23.09, 21.22) 0.9338	28.62 (-0.22, 57.45) 0.0519	1.53 (-6.79, 9.85) 0.7184	
High school or equivalent	Reference	26.75 (2.93, 50.58) 0.0279	-16.36 (-40.31, 7.59) 0.1808	-9.52 (-15.63, -3.41) 0.0023	
Some college or above	Reference	-6.95 (-18.22, 4.32) 0.2270	-4.62 (-19.28, 10.05) 0.5372	-10.42 (-13.92, -6.92) < 0.0001	
**Marital status**					0.2740
Married/living with partner	Reference	6.94 (-4.65, 18.53) 0.2405	1.15 (-14.55, 16.85) 0.8854	-7.13 (-10.82, -3.43) 0.0002	
Divorced/separated/widowed	Reference	-9.84 (-39.21, 19.53) 0.5113	-21.59 (-57.15, 13.96) 0.2340	-12.41 (-21.15, -3.67) 0.0054	
Never married	Reference	-11.64 (-25.15, 1.86) 0.0913	6.11 (-8.43, 20.66) 0.4102	-7.33 (-11.41, -3.25) 0.0004	
**PIR**					0.0828
<=1.3	Reference	28.94 (0.19, 57.69) 0.0486	-11.29 (-42.44, 19.86) 0.4774	-8.47 (-16.47, -0.48) 0.0379	
1.3–3.5	Reference	-0.69 (-13.19, 11.81) 0.9138	1.29 (-14.36, 16.93) 0.8719	-7.40 (-11.41, -3.39) 0.0003	
> 3.5	Reference	-13.16 (-24.99, -1.32) 0.0294	0.89 (-14.93, 16.71) 0.9122	-10.25 (-14.01, -6.48) < 0.0001	
**Diabetes**					0.0062
Yes	Reference	0.97 (-7.19, 9.13) 0.8158	-5.33 (-15.37, 4.72) 0.2987	-7.94 (-10.46, -5.42) < 0.0001	
No	Reference	-21.17 (-84.03, 41.69) 0.5093	75.20 (-19.06, 169.46) 0.1182	-15.89 (-36.61, 4.82) 0.1329	
**Smoking**					0.9895
Yes	Reference	-1.33 (-20.07, 17.40) 0.8893	-3.89 (-25.17, 17.39) 0.7201	-8.44 (-13.70, -3.18) 0.0017	
No	Reference	0.50 (-8.79, 9.79) 0.9153	-1.04 (-13.21, 11.13) 0.8668	-8.51 (-11.56, -5.46) < 0.0001	
**Drinking**					0.2413
No	Reference	-5.97 (-17.26, 5.33) 0.3005	0.34 (-13.14, 13.83) 0.9603	-8.65 (-12.02, -5.29) < 0.0001	
Yes	Reference	13.93 (-0.79, 28.66) 0.0638	-7.75 (-28.42, 12.91) 0.4621	-6.86 (-11.98, -1.75) 0.0086	
**CVD**					0.8475
No	Reference	-0.98 (-10.44, 8.47) 0.8387	-1.72 (-13.49, 10.05) 0.7741	-8.32 (-11.26, -5.39) < 0.0001	
Yes	Reference	13.49 (-29.28, 56.27) 0.5366	-7.38 (-61.00, 46.24) 0.7874	-12.65 (-25.51, 0.20) 0.0541	
**BMI**					0.0710
< 25	Reference	-0.16 (-5.13, 4.82) 0.9513	-3.33 (-10.23, 3.57) 0.3446	-3.96 (-5.71, -2.21) < 0.0001	
25–30	Reference	-1.59 (-16.16, 12.99) 0.8312	8.85 (-7.65, 25.36) 0.2932	-5.91 (-10.18, -1.64) 0.0067	
>=30	Reference	-0.26 (-23.82, 23.30) 0.9828	-9.19 (-37.62, 19.24) 0.5264	-14.54 (-21.31, -7.78) < 0.0001	

***Abbreviations***: PA, physical pattern; LAP, Lipid Accumulation Product; CVD, cardiovascular disease; PIR, ratio of family income to poverty.

Adjusted for age, gender, race, education, PIR, marital status, smoking, CVD, diabetes, alcoholism, and BMI.

###  Dose-response relationships between total PA and LAP

**Fig. 5:**
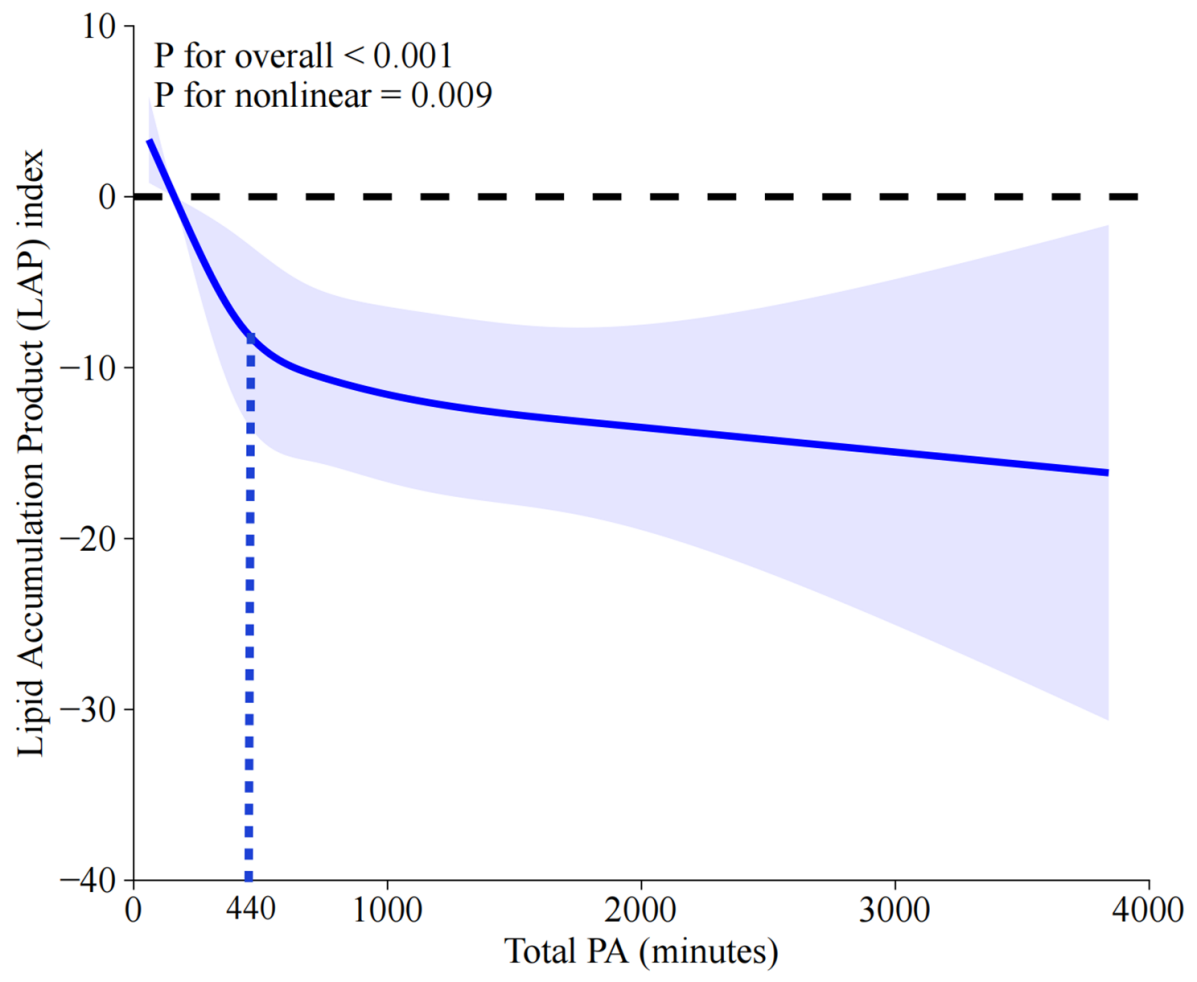
RCS analysis to explore the association between weekly total PA and LAP in all participants. Variables of age, gender, race, education, PIR, marital status, smoking, CVD, diabetes, drinking, and BMI were adjusted during RCS analyses. ***Abbreviations***: RCS, restricted cubic spline; LAP, lipid accumulation product; CVD, cardiovascular disease; BMI, body mass index; PIR, ratio of family income to poverty

RCS analyses were employed to explore the potential non-linear relationship between weekly total PA and LAP in non-inactive individuals. The results, as presented in Fig. 5, suggested a significant, non-linear, and negative correlation between weekly total PA and LAP (*P* < 0.001, P for nonlinearity = 0.009). Additionally, threshold effect and saturation effect analyses were conducted to identify the inflection point for total PA, with the results presented in Table 4. An inflection point of 440 min for weekly total PA was identified (Fig. 5) and significant differences were observed before and after the inflection point by using the bi-segmented linear regression model (effect size difference of 2 vs. 1, *P* < 0.0001). The entire group was subsequently divided into two subgroups based on the identified inflection point, individuals who performed over 440 min of PA weekly obtained significantly higher LAP reduction in all multivariate linear regression models (Table 5), with the β being − 21.516, -16.461 and − 7.759 in model 1, 2 and 3, respectively.

**Table 4:** Identification of inflection point for total PA using threshold effect and saturation effect analyses

Outcome	β (95%CI) *P*-value
***Model I***	
A straight-line effect	-0.002 (-0.004, -0.001) 0.0010
***Model II***	
Fold points (K)	440
< K-segment effect 1	-0.018 (-0.025, -0.011) < 0.0001
> K-segment effect 2	-0.001 (-0.002, 0.001) 0.3222
Effect size difference of 2 vs. 1	0.017 (0.009, 0.025) < 0.0001
Equation predicted values at break points	46.323 (43.643, 49.002)
Log likelihood ratio tests	< 0.001

Abbreviations: CI, confidence intervals; PA, physical activity; LAP, Lipid accumulation product index.

Outcome variable: LAP. Exposure variable: total PA. Adjusted for age, gender, race, education, PIR, marital status, smoking, drinking, diabetes, BMI and CVD.

**Table 5:** The relationship between total PA and LAP in all participants

Characteristics	Model 1 β (95% CI)	Model 2 β (95% CI)	Model 3 β (95% CI)
<=440	Reference	Reference	Reference
>440	-21.516 (-24.805, -18.227) < 0.00001	-16.461 (-19.824, -13.098) < 0.00001	-7.759 (-10.774, -4.744) < 0.00001

Abbreviations: PA, physical pattern; LAP, lipid accumulation product; CI, confidence interval

Model 1 was the univariate model in which no covariates were adjusted

Model 2 was adjusted for demographic covariates, including age, gender, race, education, PIR, and marital status

Model 3, based on Model 2, was additionally adjusted for smoking, CVD, diabetes, alcoholism, and BMI.

**Fig. 6:**
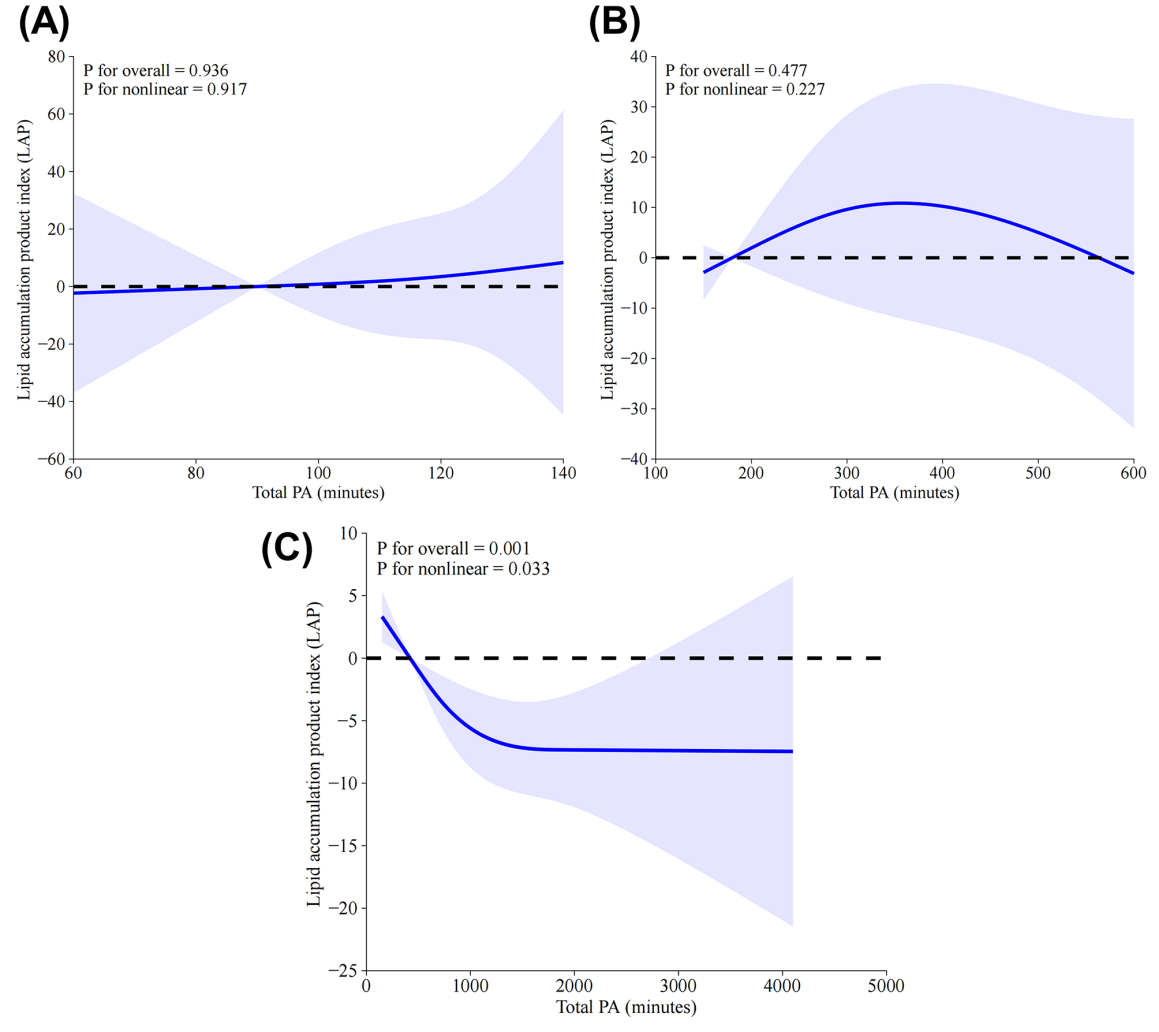
Identification of the association between weekly total PA and LAP by RCS analyses. (**A**) Insufficiently active group; (**B**) Weekend warriors group; (**C**) Regularly active group. Variables of age, gender, race, education, PIR, marital status, smoking, CVD, diabetes, drinking, and BMI were adjusted during RCS analyses. ***Abbreviations***: RCS, restricted cubic spline; LAP, lipid accumulation product; CVD, cardiovascular disease; BMI, body mass index; PIR, ratio of family income to poverty

**Fig. 7:**
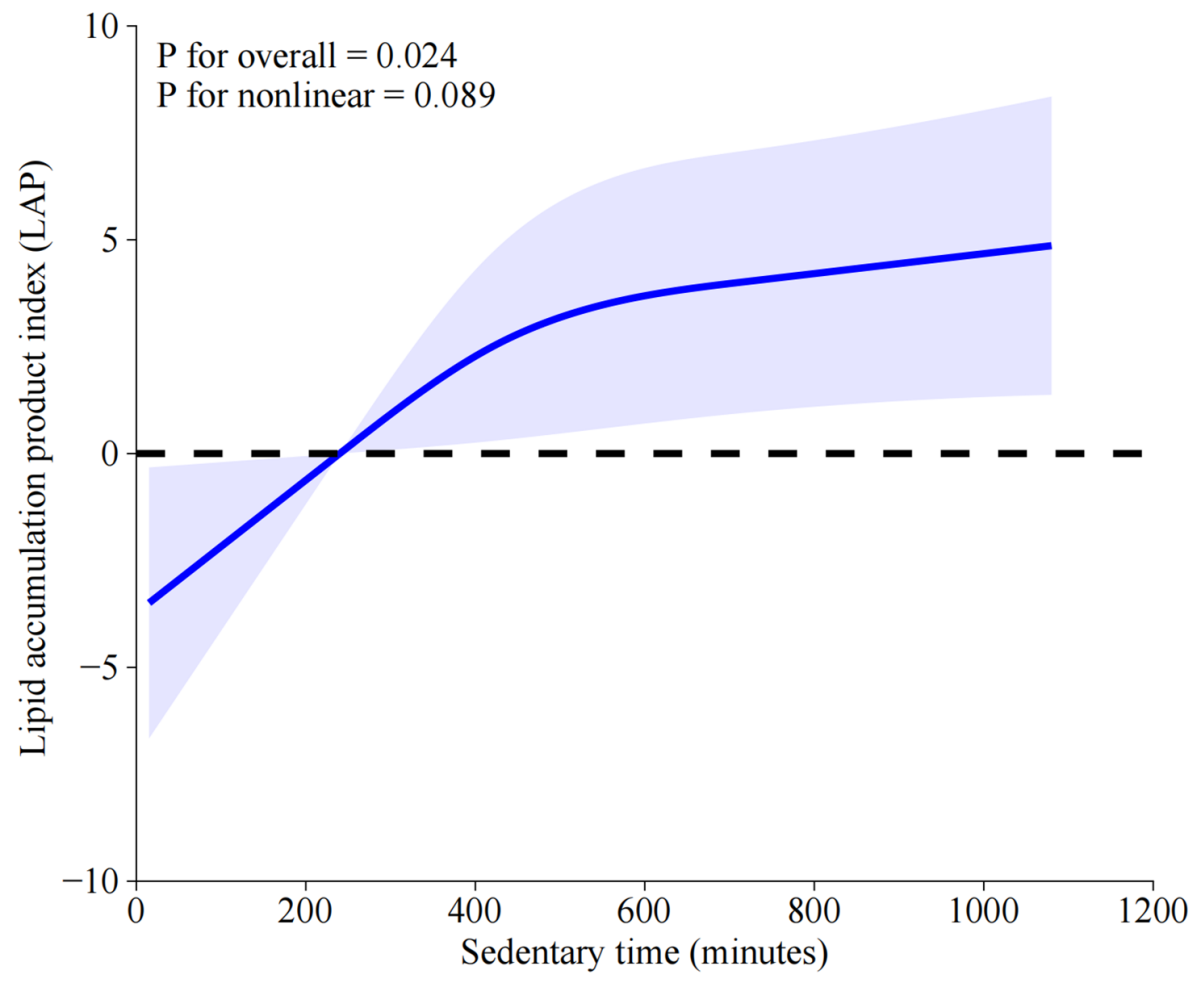
RCS analysis illustrated the association between weekly sedentary time and LAP. Variables of age, gender, race, education, PIR, marital status, smoking, CVD, diabetes, drinking, and BMI were adjusted during RCS analyses. ***Abbreviations***: RCS, restricted cubic spline; LAP, lipid accumulation product; CVD, cardiovascular disease; BMI, body mass index; PIR, ratio of family income to poverty

Except for the above-mentioned analyses, the non-linear relationship between weekly total PA and LAP in different subgroups were also performed by using RCS analyses, with the results being presented in Fig. 6. No significant and non-linear associations between weekly total PA and LAP were identified in insufficiently active group (*P* = 0.936, P for nonlinearity = 0.917; Fig. 6A) and WWs group (*P* = 0.477, P for nonlinearity = 0.227; Fig. 6B). In contrast, a negative and non-linear association between weekly total PA and LAP were found in the regularly active group (*P* = 0.001, P for nonlinearity = 0.033; Fig. 6C). As depicted in Fig. 7, the weekly sedentary time was found to be positively and linearly associated with LAP in the entire group as opposed to total PA in a week (*P* = 0.024, P for nonlinearity = 0.089).

## Discussion

In this study, we mainly focused on elucidating the association between PA patterns and LAP. Our results firstly revealed that only the PA pattern of RA was associated with significant LAP reduction as opposed to other PA patterns in the non-adjusted, partially-adjusted and fully-adjusted linear regression models. Noteworthily, being WWs didn’t harvest equivalent and significant LAP reduction as being RA. Furthermore, subgroup analyses demonstrated that the RA-LAP association remained consistent in all strata except for the variables of education and diabetes, which were proved to pose significant modification effects on the association, with the RA-LAP association being more pronounced in individuals with higher education level and diabetes. Besides, a significant, non-linear, and negative correlation between weekly total PA and LAP has been identified by RCS analysis, and an inflection point of 440 min for weekly total PA was determined by using threshold effect and saturation effect analyses, with significant differences being observed before and after the inflection point by using the bi-segmented linear regression model. Additionally, RCS analyses in subgroups suggested that significant, negative and non-linear association between weekly total PA and LAP were only found in RA group, but not in insufficiently active group or WWs group. In contrast with the weekly total PA, the weekly sedentary time was found to be significantly, positively and linearly associated with LAP in the entire group.

Previous studies have demonstrated that being WWs reaps similar healthy benefits in various aspects, including lowering the risk for incident cardiovascular events [40], reducing all-cause/CVD/cancer mortality risks [18], reducing the risk of depression symptoms [22, 30], decreasing visceral adiposity index [41], and so on. However, in this study, we discovered that only being RA got significantly LAP reduction, while being WWs did not obtain equivalent LAP reduction as expected, which is different from previous findings. Such discrepancy may be attributed to the following aspects. Since the health benefits from PA were principally depended on three PA parameters, namely frequency, duration and intensity, the differences in LAP reduction between RA group and WWs group can be analyzed from the three aspects. Firstly, previous studies have revealed that there was an minimal exercise threshold to bring about metabolic changes and thus controlling obesity. For example, exercise consuming approximately 1200 to 1600 kcal per week (i.e., performing MPA 3 to 5 times per week, equivalent to 7 ~ 14 miles) may be enough to result in favorable changes in HDL-C metabolism and thus reduced coronary heart disease mortality [42]. Therefore, although being WWs have reached total PA level of 150 min, which may be not enough to arouse changes in lipid metabolism. Actually, our findings suggested that the median of weekly total PA in WWs group was 300 min, which was obviously lower than the threshold (440 min) to reap significant LAP reduction and the median of weekly total PA in RA group (540 min). Furthermore, research conducted by Ryuki Hashida et al. demonstrated that completing exercise for a minimum of 40 ~ 45 min per session, three times a week for a duration of 12 weeks, regardless of aerobic or resistance exercise, can result in significant improvement in hepatic steatosis [43]. This suggests that engaging in exercise solely during two sessions (as WWs) may not be adequate to improve metabolic status. Additionally, an earlier study reported that the amount and frequency of PA, instead of mode and intensity of PA, was more important to affect glycaemic control in patients with type 2 diabetes [44]. Patients are advised to conduct more times of PA per week than increase the duration of per session or exercise intensity to maximize their glycaemic control. This implies that WWs may be not suitable for patients with metabolic disorders. Besides, a research performed by Suk-Yong Jang et al. suggested that being WWs (OR = 1.29, 95%CI:1.02–1.65) obtained significantly higher likelihood to develop metabolic syndrome than the RA group [23], which suggested that being WWs may arouse metabolic disorders and thus hindered the beneficial effects of PA. Finally, previous studies have suggested that continuous and long-term MPA is crucial for eliminating excess visceral fat in obese women [45], which also highlighted the importance of frequency and duration of PA in a week in reducing fat accumulation. Briefly, our results preliminary indicated that being regularly active and simultaneously reach a certain amount of PA can help to combat obesity and control LAP effectively.

In addition to the above-mentioned findings, we also found that the associations between PA patterns and LAP was modified by the variables of diabetes and education level. Participants included in the subgroup of higher education levels and complicated with diabetes showed more pronounced associations between PA pattern and LAP. Previously, amounting evidences have illustrated that patients with diabetes were usually significantly associated with higher LAP level [46], and LAP can be served as one of the effective discriminators for prediabetes and diabetes [47, 48]. Therefore, those who are obese and complicated with diabetes may obtain a higher LAP level than those who not. Given that regular exercise per se was one of the most effective interventional strategies to control diabetes and combating obesity, being RA may obtain more pronounced LAP reduction in obese individuals with diabetes. Besides, Diego Montano has reported that cardiometabolic risk was tightly associated with education differences, individuals with lower education levels got significantly higher cardiometabolic risk compared with those who were higher educated [49]. Education differences were found in multiple obesity-related indexes, including glycated haemoglobin, total cholesterol and BMI [49]. Education may modify the association between PA pattern and LAP in a rather complexed way, possibly via modulating the behavior, attitude and adaptation to PA.

According to the 2020 guidelines on PA from the World Health Organization, all adults are recommended to undertake 150 ~ 300 min of MPA, or 75–150 min of VPA, or the equivalent combination of MPA and VPA, per week [14]. The defined threshold of MVPA in 300 min for adults was based on the evidence that many of the benefits from PA began to occur when the average weekly PA volumes reach 300 min [14]. Previous studies have indicated a curvilinear dose-response association between PA volume and health outcomes, such as CVD mortality, diabetes, and so on [14]. As PA volume increases, additional benefits may be obtained, but such benefits may diminish at higher levels of PA, which is difficult to specify and it may vary in different health conditions [14]. Therefore, there is an interval between 300 min and a threshold level of PA to reap continuous improvement in health benefits. In this study, a threshold of 440 min for weekly total PA was identified by saturation and threshold effects analysis. A rapidly decreased trend in LAP levels was found as total PA increased before this inflection point (0 ~ 440), while the reduction rate of LAP remained relatively high and did not change significantly despite further increases in total PA. Therefore, it is recommended to do at least 440 minutes’ (approximately ≥ 62 min/daily) MVPA per week to achieve favorable LAP reduction. The results from our study, combined with the 2020 PA guidelines from the World Health Organization, have demonstrated that a minimal PA of 300 min per week is needed to keep one’s fundamental fitness, but additional PA is required in order to achieve a specific health goal (e.g., combating obesity or controlling LAP).

Our study possesses several advantages. It represents the first investigation to examine the correlation between the obesity-specific evaluation index LAP and various PA patterns. Stratified analyses, combined with the interaction tests were employed the robustness of PA-LAP association. Additionally, we innovatively employed RCS curves to illustrate the non-linear relationship between total PA and LAP. However, some limitations should be acknowledged in this study. Firstly, due to the cross-sectional analysis, causal relationships between different PA patterns and LAP cannot be deduced. Secondly, the self-reported measurement of PA may be not as accurate as the measurement of PA via accelerometry for the reason that self-reports may overestimate duration and intensity of PA, as reported in previous studies [50–52]. Thirdly, although various potential confounding factors have been adjusted in this study, the possibility of unmeasured confounding factors cannot be entirely eliminated. Finally, integrated analyses between PA parameters (including duration, session and intensity) and other indicators from high-throughput omics, such as metabolomics, microbiomics, proteomics, and so on, are recommended to get in-depth knowledge about the influences of PA on human fitness.

In summary, our findings from the current study highlighted the critical role of PA in combating obesity and controlling LAP. Being regularly active, in comparison with being weekend warriors throughout the week, was associated with significant and favorable LAP reduction. Moreover, engaging in more than 440 min of physical activity per week helped to yield optimal benefits in terms of reducing LAP.

### Electronic supplementary material

Below is the link to the electronic supplementary material.

Supplementary Material 1

## Data Availability

Publicly available datasets were analyzed in this study. This data can be accessible at: https://www.cdc.gov/nchs/nhanes/index.htm.

## Abbreviations

BMI: body mass index
CVD: cardiovascular diseases
LAP: Lipid accumulation product
MPA: moderate physical activity
MVPA: moderate-to-vigorous physical activity
PA: physical activity
PIR: poverty income ratio
RA: regular active
RCS: restricted cubic spline
TG: triglycerides
VPA: vigorous physical activity
WC: waist circumference
WWs: weekend warriors
