# Supplementary material for: Whether weekend warriors (WWs) achieve equivalent benefits in lipid accumulation products (LAP) reduction as other leisure-time physical activity patterns? -Results from a population-based analysis of NHANES 2007–2018
Source: BMC Public Health. 2024 Jun 9;24:1550. doi: 10.1186/s12889-024-19070-z (PMC11163723; doi:10.1186/s12889-024-19070-z)
Supplement: Supplementary file 1 — Supplementary Material 1 [file 12889_2024_19070_MOESM1_ESM.docx]

**Supplementary Table 1. The characteristics of physical activity among different PA pattern groups.**

| **Characteristics** | **Overall** | **Inactive** | **Insufficiently active** | **Weekend warriors** | **Regularly active** | **P-value*** |
| --- | --- | --- | --- | --- | --- | --- |
| Sedentary time, minutes | 300 (180-480) | 300 (180-480) | 360 (180-540) | 300 (180-480) | 300 (180-480) | 0.334 |
| VPA sessions per week, times | 3 (2-4) | 0 (0-0) | 1 (1-2) | 1 (1-1) | 3 (2-4) | <0.001 |
| VPA time per session, minutes | 60 (40-90) | 0 (0-0) | 30 (15-30) | 75 (60-120) | 60 (42-90) | <0.001 |
| Total VPA, minutes | 360 (210-600) | 0 (0-0) | 60 (60-90) | 150 (120-240) | 360 (240-600) | <0.001 |
| MPA sessions per week, times | 3 (2-5) | 0 (0-0) | 2 (1-2) | 1 (1-1) | 3 (2-5) | <0.001 |
| MPA time per session, minutes | 60 (30-60) | 0 (0-0) | 30 (15-30) | 90 (60-120) | 60 (30-60) | <0.001 |
| Total MPA, minutes | 135 (90-240) | 0 (0-0) | 30 (30-60) | 90 (60-120) | 150 (90-240) | <0.001 |
| Total MVPA sessions, times | 6 (5-8) | 0 (0-0) | 3 (2-4) | 2 (2-2) | 6 (5-8) | <0.001 |
| Total PA, minutes | 540 (330-840) | 0 (0-0) | 120 (90-140) | 300 (180-360) | 540 (360-900) | <0.001 |

***Abbreviations***: VPA, vigorous physical activity; MPA, moderate physical activity; MVPA, moderate-to-vigorous physical activity. ***Note****:* Variables were presented as Median (Q1-Q3).

**Supplementary Table 2. The relationship between physical activity pattern and LAP.**

| **Characteristics** | **Model 1 β (95% CI)** | **Model 2 β (95% CI)** |
| --- | --- | --- |
| **PA pattern** |  |  |
| Inactive | Reference | Reference |
| Insufficiently active | -5.52 (-14.39, 3.35) 0.2227 | -9.13 (-19.30, 1.04) 0.0784 |
| Weekend warrior | -4.70 (-15.29, 5.89) 0.3841 | -8.90 (-21.57, 3.78) 0.1690 |
| Regularly active | -8.85 (-11.59, -6.11) <0.0001 | -17.15 (-20.29, -14.00) <0.0001 |
| P for trend | <0.001 | <0.001 |
| **PA pattern** |  |  |
| Regularly active | Reference | Reference |
| Inactive | 8.85 (6.11, 11.59) <0.0001 | 17.15 (14.00, 20.29) <0.0001 |
| Insufficiently active | 3.33 (-5.55, 12.20) 0.4626 | 8.02 (-2.21, 18.24) 0.1243 |
| Weekend warrior | 4.14 (-6.47, 14.75) 0.4442 | 8.25 (-4.47, 20.97) 0.2035 |
| P for trend | <0.001 | <0.001 |

***Abbreviations***: PA, physical pattern; LAP, lipid accumulation product; CI, confidence interval.

***Model 1*** was adjusted for age, gender, race, education, PIR, marital status, smoking, CVD, diabetes, drinking, and BMI.

***Model 2*** was adjusted for age, gender, race, education, PIR, marital status, smoking, and drinking (variables of CVD, diabetes and BMI were excluded for adjustment).
